# Supplementary material for: Culturomics Approach to Identify Diabetic Foot Infection Bacteria
Source: Int J Mol Sci. 2021 Sep 3;22(17):9574. doi: 10.3390/ijms22179574 (PMC8431627; doi:10.3390/ijms22179574)
Supplement: Supplementary file 1 [file ijms-22-09574-s001.zip › ijms-1304799-supplementary.pdf]

		COL	MHA	TSA	BHI	BLA	CHRA	BCP	AZI	MAN	VRE
Gram-positive	***Corynebacterium striatum***	2.42 ± 0.07	2.30 ± 0.01	2.38 ± 0.07	2.36 ± 0.05	1.96 ± 0.20	2.20 ± 0.15	2.34 ± 0.07	-	-	2.33 ± 0.17
	Enterococcus faecalis	2.16 ± 0.10	2.19 ± 0.13	2.01 ± 0.22	2.09 ± 0.11	2.19 ± 0.11	2.12 ± 0.12	2.14 ± 0.12	2.10 ± 0.15	2.21 ± 0.06	2.12 ± 0.16
	Streptococcus agalactiae	2.27 ± 0.12	2.32 ± 0.02	2.15 ± 0.18	2.25 ± 0.13	2.32 ± 0.09	2.26 ± 0.11	2.28 ± 0.06	2.26 ± 0.02	-	2.14 ± 0.10
	Streptococcus pyogenes	2.18 ± 0.11	2.22 ± 0.10	2.30 ± 0.11	2.20 ± 0.07	2.32 ± 0.14	2.39 ± 0.06	2.36 ± 0.05	2.11 ± 0.18	-	-
	Staphylococcus aureus	2.27 ± 0.15	2.04 ± 0.17	2.22 ± 0.12	2.12 ± 0.19	2.15 ± 0.13	2.10 ± 0.19	2.04 ± 0.13	2.24 ± 0.09	2.06 ± 0.17	2.11 ± 0.21
	Streptococcus dysgalactiae	2.14 ± 0.15	2.27 ± 0.07	2.30 ± 0.08	2.25 ± 0.05	2.09 ± 0.07	-	2.33 ± 0.06	-	-	2.22 ± 0.06
	Staphylococcus epidermidis	-	-	-	-	-	-	-	-	1.78 ± 0.07	-
	Staphylococcus haemolyticus	2.04 ± 0.08	2.14 ± 0.15	2.17 ± 0.13	1.99 ± 0.11	2.11 ± 0.12	1.93 ± 0.12	2.05 ± 0.07	2.46 ± 0.14	1.86 ± 0.12	1.98 ± 0.17
	Staphylococcus simulans	1.81 ± 0.01	-	-	-	1.77 ± 0.02	1.85 ± 0.08	-	-	-	2.01 ± 0.01
	Helcococcus kunzii	-	-	-	-	2.24 ± 0.08	-	-	-	-	2.41 ± 0.03
Gram-negative	***Escherichia coli***	-	-	-	-	-	2.07 ± 0.11	2.00 ± 0.18	-	-	-
	Klebsiella oxytoca	1.87 ± 0.14	1.86 ± 0.07	-	1.95 ± 0.14	2.00 ± 0.11	1.94 ± 0.13	1.96 ± 0.12	-	-	-
	Citrobacter freundii	2.24 ± 0.10	2.08 ± 0.08	2.08 ± 0.17	2.01 ± 0.15	2.18 ± 0.14	2.03 ± 0.10	1.89 ± 0.07	-	-	2.33 ± 0.09
	Enterobacter cloacae	1.97 ± 0.17	-	1.79 ± 0.05	-	-	2.05 ± 0.17	-	-	-	-
	Morganella morganii	-	-	-	-	1.79 ± 0.05	2.17 ± 0.17	2.37 ± 0.07	-	-	2.48 ± 0.05
	Proteus mirabilis	2.13 ± 0.23	2.06 ± 0.09	2.17 ± 0.15	2.22 ± 0.11	1.89 ± 0.14	2.10 ± 0.11	2.13 ± 0.21	-	-	2.35 ± 0.04
	Proteus vulgaris	-	-	-	-	1.78 ± 0.09	1.92 ± 0.04	2.05 ± 0.09	-	2.26 ± 0.07	-
	Pseudomonas aeruginosa	2.05 ± 0.14	2.19 ± 0.14	2.07 ± 0.12	2.02 ± 0.20	1.96 ± 0.13	1.99 ± 0.16	2.00 ± 0.17	-	-	2.04 ± 0.16

Table S1 Impact of the culture media composition on the identification level of bacteria expressed as score value (mean + SD) obtained using MALDI Biotyper 3.0 Platform.

Patient	Gender	Age
P1	Male	80 yrs.
P2	Female	63 yrs.
P3	Male	69 yrs.
P4	Female	85 yrs.
P5	Female	87 yrs.
P6	Male	74 yrs.
P7	Male	57 yrs.
P8	Male	73 yrs.
P9	Female	68 yrs.
P10	Male	62 yrs.
P11	Male	54 yrs.
P12	Male	53 yrs.
P13	Male	81 yrs.
P14	Female	65 yrs.
P15	Male	71 yrs.
P16	Male	65 yrs.

Table S2 List of investigated DFI patients with information about gender and age.

m/z	VRE	BLA	Protein
			C. striatum
3215.6	+	-	Transposase
4688.4	-	+	50S ribosomal protein L36
6232.2	-	+	Amino acid dehydrogenase
6880.9	-	+	Transposase-like protein/Two-component system response regulator
7231.6	-	+	50S ribosomal protein L35
7245.9	-	+	50S ribosomal protein L35
10316.7	+	-	30S ribosomal protein S15
10595.7	+	-	Plasmid stabilization protein
11191.8	+	-	50S ribosomal protein L24
11684.1	-	+	Protein translocase subunit SecE
11707.4	-	+	DNA topoisomerase (ATP-hydrolyzing)
			P. mirabilis
m/z	**VRE**	**BLA**	**Protein**
4372.6	-	+	Glucose-6-phosphate isomerase
4484.9	-	+	50S ribosomal protein L36
4668.4	-	+	RNase E inhibitor protein
4757.0	+	-	3-deoxy-D-manno-octulosonate 8-phosphate phosphatase
5026.6	+	-	Protein of uncharacterized function (DUF2857)
5110.4	+	-	Arylsulfatase
5120.3	+	-	Cytoplasmic protein
5365.8	-	+	Peroxidase
5382.0	-	+	Rho-binding antiterminator
5456.1	+	-	Integrase
5496.4	+	-	IrpP
5778.0	-	+	Single-stranded DNA-binding protein
6007.6	-	+	ATP-dependent helicase HepA
6032.7	-	+	TdRPase
6052.8	-	+	LysR-family transcriptional regulator
6096.3	-	+	Spermidine/putrescine ABC transporter membrane protein
6105.6	-	+	Isocitrate dehydrogenase (NADP(+))
6122.2	-	+	YtxH domain-containing protein
6255.1	-	+	Fimbrial subunit
6281.1	+	-	ABC transporter substrate-binding protein
6468.2	+	-	N-acetylmuramoyl-L-alanine amidase AmiC
6492.9	-	+	Ribosome modulation factor
6858.2	-	+	UPF0434 protein PMI0721
7138.8	+	-	Phage protein
7142.7	-	+	Fimbrial protein
7178.4	-	+	Holin
7233.4	+	-	Plasmid-like protein
7291.9	+	-	IS element transposase
7295.3	-	+	Fimbrial chaperone
7829.2	-	+	50S ribosomal protein L31
7830.1	+	-	Lipoprotein

7979.3	-	+	Aminomethyl transferase family protein
8021.4	-	+	KTSC domain-containing protein
8043.1	-	+	Inner membrane transport protein Yha
8329.1	+	-	Glutaredoxin-like protein NrdH
8356.6	-	+	Protein SlyX
8358.6	+	-	Coproporphyrinogen III oxidase
8393.6	-	+	Deoxyribose-phosphate aldolase
8826.0	-	+	Adenosine-3'(2'),5'-bisphosphate nucleotidase
8846.8	-	+	Trehalose repressor
8906.5	-	+	Ribosome modulation factor
8927.0	-	+	Acyl-CoA thioesterase/SirA-like protein
8999.0	+	-	Lipoprotein
9018.7	+	-	Fimbrial chaperone protein
9448.0	-	+	Uncharacterized N-acetyltransferase YjaB
9471.8	-	+	Cyclic di-GMP-binding protein
9474.2	+	-	Pyocin activator protein PrtN
9509.3	-	+	Ig-like domain-containing protein
9512.7	+	-	Putative phage protein
9553.1	+	-	Carbon dioxide concentrating mechanism protein CcmL
9574.5	-	+	Spermidine export protein MdtJ
9589.2	-	+	Putative membrane protein insertion efficiency factor
9591.3	+	-	Sec-independent protein translocase protein TatA
9608.0	-	+	Peptide permease
10219.2	+	-	Putative DNA-binding transcriptional regulator
10286.9	+	-	Na(+)-translocating NADH-quinone reductase subunit D
10988.5	+	-	DNA topoisomerase (ATP-hydrolyzing)
11002.0	-	+	7,8-dihydroneopterin aldolase
11004.7	+	-	Insulinase family protein
			M. morganii
m/z	**VRE**	**BLA**	**Protein**
3094.3	+	-	Potassium-transporting ATPase subunit F
3373.0	+	-	Phage tail protein
3744.2	-	+	Diacylglycerol kinase
5333.4	+	-	Heat shock protein J
5395.9	+	-	50S ribosomal protein L34
5497.8	-	+	IrpP
6202.6	+	-	CAAX protease
6232.5	+	-	Lysophospholipase
6386.4	+	-	Ribosome modulation factor
6536.9	-	+	TetR_C_1 domain-containing protein
6614.2	+	-	50S ribosomal protein L30
6919.8	+	-	DUF2526 protein
6929.7	-	+	Transposase
7067.8	-	+	Lipoprotein/?
7108.6	-	+	Sulfur carrier protein ThiS
7132.9	-	+	Holin

7148.3	-	+	DUF3761 domain-containing protein
7197.5	+	-	Holin
7208.4	+	-	Erythromycin esterase/Hemolysin expression modulating protein
7300.9	+	-	TetR family transcriptional regulator
7328.4	+	-	DNA polymerase I
7742.2	-	+	DUF4222 domain-containing protein
7928.0	-	+	DNA-binding protein/XRE family transcriptional regulator
8298.7	+	-	Probable [Fe-S]-dependent transcriptional repressor
8326.4	-	+	DUF1471 domain-containing protein
8340.2	+	-	Putative cI repressor protein
8410.8	-	+	Protein SlyX
8669.8	+	-	Acyl carrier protein
8702.0	-	+	TIGR03758 family integrating conjugative element protein
8814.6	+	-	Acyl-CoA dehydrogenase
8829.5	+	-	2Fe-2S ferredoxin-like protein
8887.0	-	+	YajA protein
8979.3	+	-	zinc_ribbon_2 domain-containing protein
9268.3	+	-	DNA-binding protein
9279.7	-	+	Putative zinc finger/helix-turn-helix protein, YgiT family
9282.4	+	-	DNA-binding protein HU-beta
9449.6	-	+	DNA-binding protein HU-alpha
9465.6	+	-	DNA-binding protein HU-alpha
9492.1	-	+	Outer membrane protein W
9589.9	-	+	Phage tail assembly protein T
9924.0	+	-	DUF2798 domain-containing protein
10258.4	-	+	HTH cro/C1-type domain-containing protein
10271.3	-	+	Acetolactate synthase 2 regulatory subunit
10664.7	+	-	N-acetyltransferase/XRE family transcriptional regulator
11056.9	-	+	DUF2190 family protein

Table S3 Summary of signals observed on MALDI-TOF MS spectra with characterized *m*/*z* values that differed between VRE and BLA culture medium, according to the database of Universal Protein (UniProt). Table does not include signals from uncharacterized proteins

A

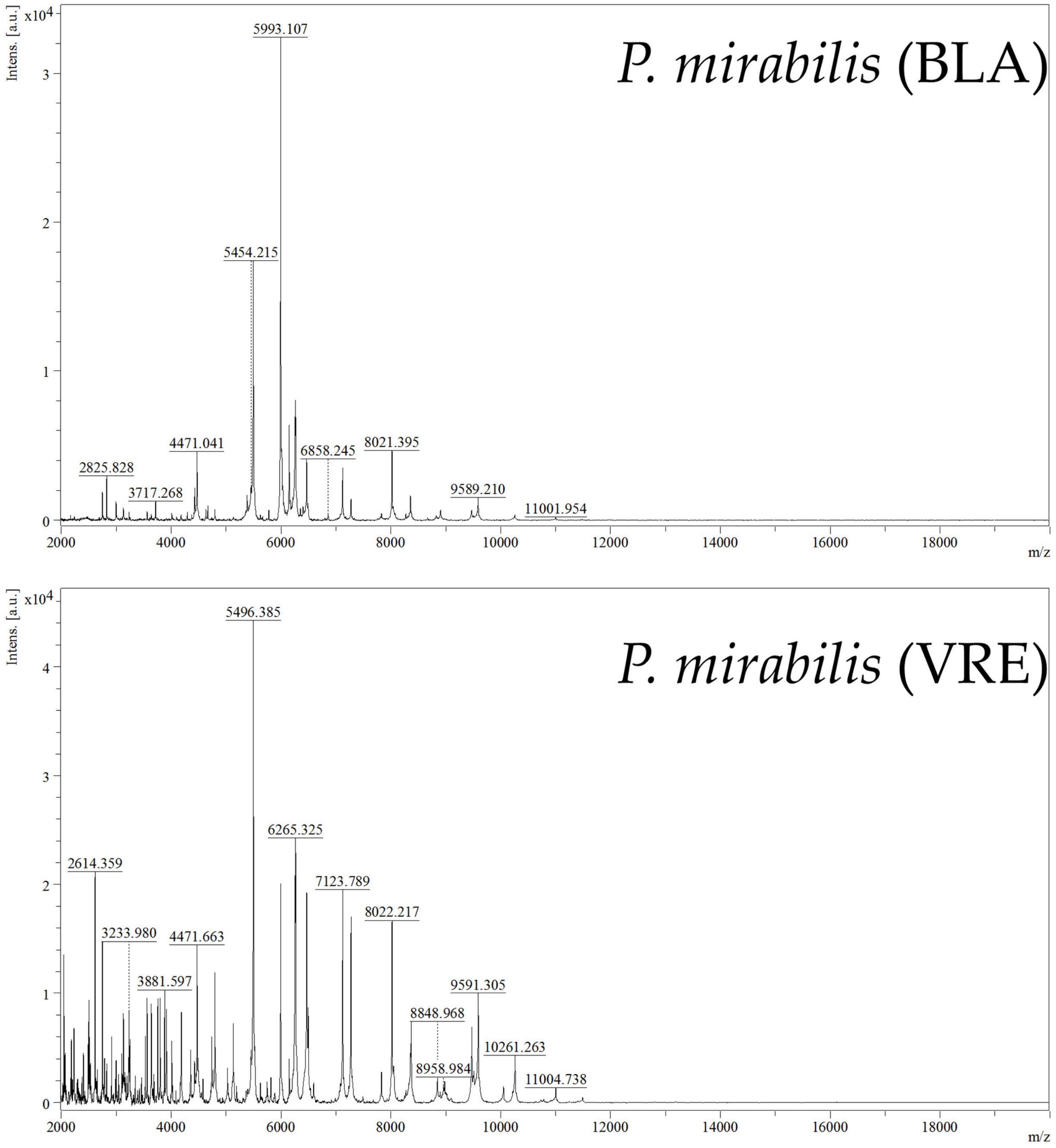

P. mirabilis (BLA)
Intens. [a.u.]
x10^4
5993.107
5454.215
4471.041
2825.828
3717.268
6858.245
8021.395
9589.210
11001.954
m/z
P. mirabilis (VRE)
Intens. [a.u.]
x10^4
5496.385
6265.325
2614.359
7123.789
8022.217
3233.980
4471.663
3881.597
9591.305
8848.968
10261.263
8958.984
11004.738
m/z

B

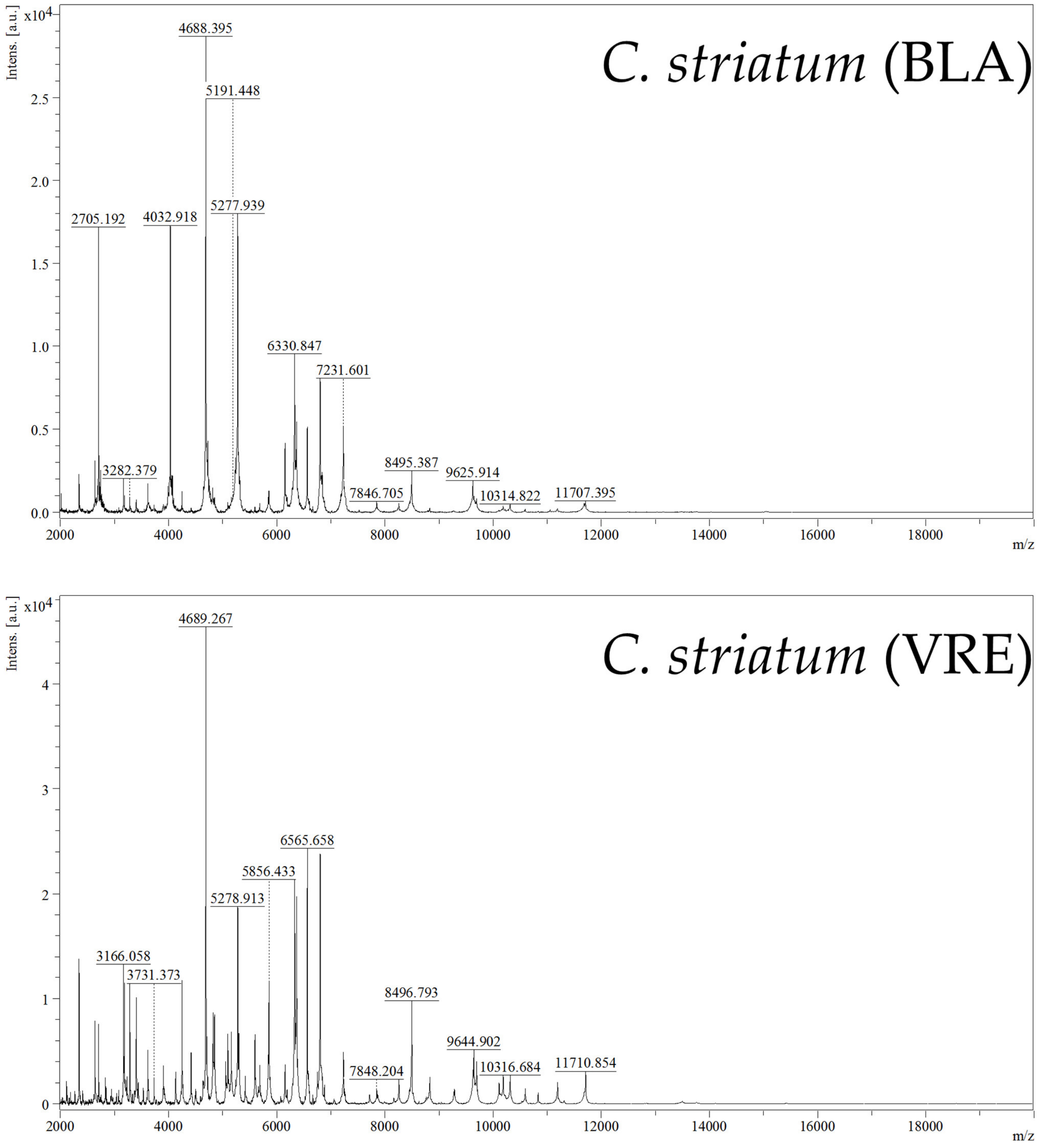
C. striatum (BLA)
Intens. [a.u.]
x10^4
4688.395
5191.448
2705.192
4032.918
5277.939
6330.847
7231.601
3282.379
8495.387
7846.705
9625.914
10314.822
11707.395
m/z
C. striatum (VRE)
Intens. [a.u.]
x10^4
4689.267
6565.658
5856.433
5278.913
3166.058
3731.373
8496.793
9644.902
10316.684
11710.854
7848.204
m/z

C

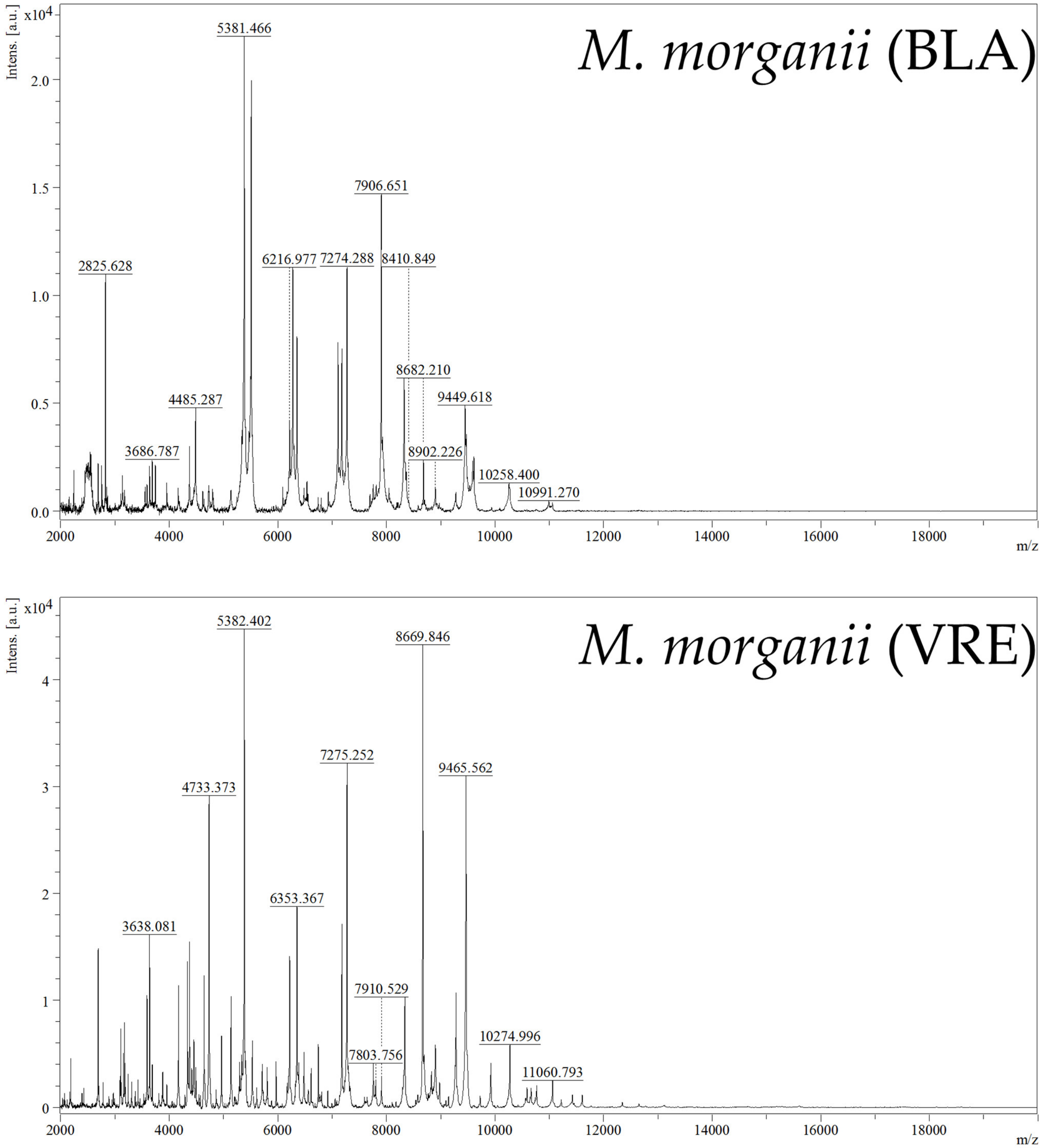

Figure S1 Comparison of the molecular profiles of the (A) *P. mirabilis*, (B) *C. striatum*, and (C) *M. morganii* cultured on the Columbia blood agar (BLA) and vancomycin resistant enterococci agar (VRE) generated during MALDI-TOF MS analysis and analyzed using FlexAnalysis software.
